# The calibration of esophageal pressure by proper esophageal balloon filling volume: A clinical study

**DOI:** 10.3389/fmed.2022.986982

**Published:** 2022-12-20

**Authors:** Jing Jiang, Longxiang Su, Wei Cheng, Chunfu Wang, Xi Rui, Bo Tang, Hongmin Zhang, Huaiwu He, Yun Long

**Affiliations:** ^1^Department of Critical Care Medicine, Peking Union Medical College, Chinese Academy of Medical Sciences, State Key Laboratory of Complex Severe and Rare Disease, Peking Union Medical College Hospital, Beijing, China; ^2^Department of Critical Care Medicine, Chongqing General Hospital, Chongqing, China; ^3^Department of Infectious Diseases, Tangdu Hospital, Air Force Medical University, Xi’an, Shaanxi, China

**Keywords:** esophageal pressure, esophageal balloon catheter, balloon filling volume, mechanical ventilation, calibration

## Abstract

**Background:**

Esophageal pressure (Pes) can be used as a reliable surrogate for pleural pressure, especially in critically ill patients requiring personalized mechanical ventilation strategies. How to choose the proper esophageal balloon filling volume and then find the optimal value of esophageal pressure remains a challenge. The study aimed to assess the feasibility of catheters for Pes monitoring in mechanically ventilated patients.

**Materials and methods:**

Twelve patients under pressure-controlled mechanical ventilation were included in this study. Raw esophageal pressure was recorded at different balloon filling volumes. Then, the P-V curves were determined. V_*WORK*_ was the intermediate linear section on the end-expiratory P-V curve, and V_*BEST*_ was the filling volume providing the maximum difference between Pes at end-inspiration and end-expiration. The raw value of Pes was recorded, and the calibrated values of Pes were calculated by calculating the esophageal wall pressure (Pew) and esophageal elastance (Ees).

**Results:**

Twenty-four series of Pes measurements were performed. The mean V_*MIN*_ and V_*MAX*_ were 2.17 ± 0.49 ml (range, 1.0–3.0 ml) and 6.79 ± 0.83 ml (range, 5.0–9.0 ml), respectively, whereas V_*BEST*_ was 4.69 ± 0.16 ml (range, 2.0–8.0 ml). Ees was 1.35 ± 0.51 cm H_2_O/ml (range, 0.26–2.38 cm H_2_O/ml). The estimated Pew at V_*BEST*_ was 3.16 ± 2.19 cm H_2_O (range, 0–7.97 cm H_2_O). Patients with a body mass index (BMI) ≥ 25 kg/m^2^ had a significantly lower V_*MAX*_ (5.88 [5.25–6] vs. 7.25 [7–8] ml, *p* = 0.006) and a significantly lower V_*BEST*_ (3.69 [2.5–4.38] vs. 5.19 [4–6] ml, *p* = 0.036) than patients with a BMI < 25 kg/m^2^. Patients with positive end-expiratory pressure (PEEP) ≥ 10 cm H_2_O had a lower V_*MIN*_ and V_*BEST*_ than patients with PEEP < 10 cm H_2_O, *P* > 0.05. Patients in the supine position had a higher esophageal pressure than those in the prone position with the same balloon filling volume.

**Conclusions:**

Calibration of esophageal pressure to identify the best filling volume of esophageal balloon catheters is feasible. The esophageal pressure can be influenced by BMI, PEEP, and position. It is necessary to titrate the optimal inflation volume again when the PEEP values or the positions change.

## Introduction

An increasing number of clinicians have been focusing on esophageal pressure (Pes) manometry because of its vital role in understanding pulmonary pathophysiology since the end of the 19th century. Due to the impossibility of directly measuring pleural pressure in clinical practice, esophageal pressure has been proposed as a reliable surrogate for pleural pressure (Ppl) (1–3). We can estimate Ppl and hence transpulmonary pressure (P_*L*_), which is the distending pressure of the lungs. In the past 20 years, this technique has been used in critically ill patients, especially patients with acute respiratory failure (ARF).

It is extremely useful to understand each patient’s individual respiratory physiology, particularly for patients with morbid obesity and acute respiratory distress syndrome (ARDS) (4, 5). First, it is useful to characterize the respiratory system mechanics during passive mechanical ventilation, such as titration of positive end-expiratory pressure (PEEP) and monitoring transpulmonary driving pressure (ΔP_*L*_). Second, it can be used to monitor patients’ respiratory muscle activity during assisted ventilation. Last, it contributes to assessing patient-ventilator interaction (i.e., synchrony and asynchrony) at bedside. Therefore, esophageal pressure can be monitored during the entire process of mechanical ventilation, especially personalized mechanical ventilation strategies (6). The esophageal pressure was described by Luciani L more than 100 years ago (1), however, esophageal manometry is still not widespread. The LUNG SAFE study showed that esophageal pressure is monitored in <1% of ARDS patients receiving invasive therapy (7, 8). It is difficult to monitor in clinical practice because the quality, accuracy, and reliability of the measurement can be affected by the characteristics of the balloon catheter, the balloon-filling pressure, the position of the catheter in the patient, and the position of the esophagus’ (7). In recent years, several researchers have focused on balloon-filling volume selection in esophageal pressure *in vitro* and *in vivo* studies. Accurate measurement of esophageal pressure was found to be clearly correlated with the balloon-filling volume. Pes can be underestimated because of underfilled balloon volume or overestimated due to overfilled balloon volume (9–13). However, the range of appropriate filling volumes varies among catheters. Any catheter needs to be verified for reliable esophageal pressure measurement by finding the optimal filling volume of the balloon. This study used Mindray second-generation balloon catheters to investigate the quality and accuracy of the catheter for Pes monitoring in mechanically ventilated patients and to analyze related factors. A fast and practical cannulation procedure is provided.

## Materials and methods

The study was approved by the Institutional Research and Ethics Committee of the Peking Union Medical College Hospital (NO. ZS-2458). Informed consent was obtained as required before data were included in the study.

We enrolled heavily sedated ICU patients (Richmond Agitation-Sedation Scale score ≤−3) with ARF under controlled mechanical ventilation. ARF was by a ratio of partial pressure of arterial oxygen to fraction of inspired oxygen less than 300 mmHg. Exclusion criteria were as follows: (1) age under 18 years; (2) any contraindication for esophageal balloon catheter insertion (diagnosed or suspected esophageal varices, history of esophageal or gastric surgery, evidence of severe coagulopathy, etc.); (3) evidence of active air leakage from the lung, including bronchopleural fistula, and pneumomediastinum; and (4) lack of informed consent.

While undergoing treatment, the included patients remained in the supine position without elevating the head of the bed. The esophageal balloon had a length of 10 cm and a nominal volume of 10 ml. The tube with esophageal and gastric balloon (SDY-2, AMK Medical, Guangzhou, China) was inserted in the mid-lower third of the thoracic esophagus for clinical purposes. Appropriate catheter position was confirmed by cardiac oscillations on Pes tracing and radiopaque markers on chest X-ray. A positive pressure occlusion test was performed at end-expiratory occlusion, and the ratio of changes in Pes to changes in Paw (ΔPes/ΔPaw) during the compression of the chest wall was calculated and maintained at 0.8∼1.2. To obtain esophageal balloon pressure-volume curves, we filled the Mindray esophageal balloon with air in 1 ml increments from 0 to 10 ml unless the esophageal pressure clearly rose. At each volume step, the balloon was completely deflated by applying a negative pressure, fully inflated with 10 ml of air, and finally deflated. Then dynamic pressures were obtained at end-inspiration or end-expiration (the esophageal pressure monitoring procedure is shown in Supplementary Video 1).

We recorded the Pes at end-inspiration (PesEI) and end-expiration (PesEE) for each filling volume in each patient. From those data, we obtained two curves to express the patient pressure-volume (P-V) relationship between balloon filling volume and esophageal balloon pressure of end-inspiration and end-expiration. On the end-expiratory P-V curve, the intermediate linear section was identified as V_*WORK*_, and the lower and upper limits were expressed as minimum and maximum filling volumes (V_*MIN*_ and V_*MAX*_). The filling volume providing the maximum difference between PesEI and PesEE was identified as V_*BEST*_. The slope of the intermediate linear section on the end-expiratory P-V curve obtained by least square fitting was defined as the elastance of the esophagus (Ees) (14). As Milic-Emili et al. (3) and Francesco Mojoli et al. (9) said, the esophageal wall pressure (Pew), for any filling volume (V_*X*_) above V_*MIN*_, was calculated as: Pew = (V_*X*_–V_*MIN*_) × Ees. The calibrated values of Pes (Pes_*CAL*_) were obtained by measuring the end-expiration Pes of the best filling volume and Pes_*CAL*_ = Pes-Pew (3, 14).

To obtain gastric balloon pressure (Pga), the Mindray gastric balloon was completely deflated by applying a negative pressure, fully inflated with 10 ml of air, and finally withdrawn by 5 ml. Then, the Pga of end-inspiration and end-expiration was recorded.

### Statistics

Patient demographics and relevant clinical data are expressed as the mean and standard deviation (SD) or median (25th–75th percentile) for continuous, variables, and numbers (percentages) for categorical variables. Differences between body mass index (BMI), position, and PEEP groups were compared by using the *t*-test or the Wilcoxon signed-rank test where appropriate. Bland-Altman analysis was used to verify the consistency of Pes_*VBEST*_ and Pes_*CAL*_ at V_*BEST*_. Upper and lower limits of agreement were defined as bias ± 1.96 SD of the mean. Statistical analyses were computed using GraphPad Prism 8.0.2 software (GraphPad Software, San Diego, CA, USA).

## Results

### Population characteristics and ventilator and respiratory parameters

Twenty-four series of measurements were collected from 12 patients (age, 48.9 ± 19.9 years, 66.7% male) under pressure-controlled mechanical ventilation. The baseline characteristics are reported in Table 1. Four patients were measured twice, one patient was measured three times, and one patient was measured five times at different ventilator parameters or underlying different disease states. Twenty-one series of measurements were taken with the patient in the supine position with the bed at 0 degrees while three series of measurements were taken with the patient in the prone position. Three series were taken with the patient receiving mechanical ventilation and VV ECMO treatment. Four series were taken where the patient had undergone thoracotomy. Eighteen series were measured for Pes and gastric internal pressure (Pga). The baseline scores of APACHE II and SOFA were 20.8 ± 7.1 and 12.4 ± 2.9, respectively. We recorded a PaO_2_/FiO_2_ of 228.6 ± 86.6 mmHg, a tidal volume/predicted body weight of 5.9 ± 1.1 ml/kg, Pplat of 21.0 ± 3.9 cm H_2_O, Pdriv of 11.5 ± 2.8 cm H_2_O, and Crs of 34.1 ± 12.2 ml/cm H_2_O.

**TABLE 1:** Baseline characteristics of the patients (*n* = 12).

Characteristic	Value
Age, years	48.9 ± 19.9
Male, *n* (%)	8 (66.7%)
Body mass index, kg/m^2^	25.8 ± 4.7
APACHE II score	20.8 ± 7.1
SOFA score	12.4 ± 2.9
PaO_2_/FiO_2_, mmHg	228.6 ± 86.6
Tidal volume/PBW, ml/kg	5.9 ± 1.1
PEEP, cm H_2_O	9.6 ± 2.8
Pplat, cm H_2_O	21.0 ± 3.9
Pdriv, cm H_2_O	11.5 ± 2.8
*Crs*, ml/cm H_2_O	34.1 ± 12.2

APACHE, acute physiology and chronic health evaluation; SOFA, sequential organ failure assessment; PaO_2_/FiO_2_: ratio of partial pressure of arterial oxygen to fraction of inspired oxygen; PBW: predicted body weight; PEEP: positive end-expiratory pressure; Pplat: airway plateau pressure; Pdriv: airway driving pressure; Crs: respiratory system compliance; Continuous data are shown as the mean value ± standard deviation.

### Parameters of end-expiratory and end-inspiratory P-V curves

**FIGURE 1:**
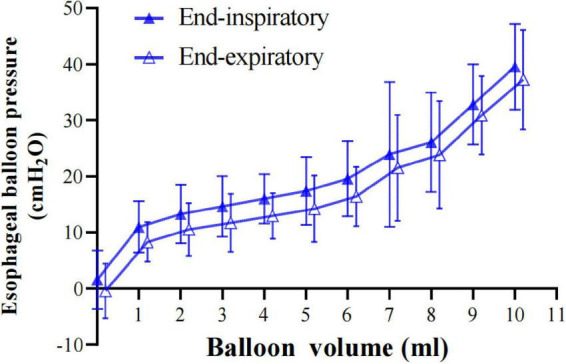
Esophageal balloon pressure-volume curves. Relationship between balloon filling volume and Pes values, both at end-inspiration (PesEI, solid blue triangle) and at end-expiration (PesEE, hollow blue triangle). On the end-expiratory pressure-volume (P-V) curve, the intermediate linear section was graphically detected and analyzed for its lower and upper limits (V_*MIN*_ and V_*MAX*_, respectively). The range between V_*MIN*_ and V_*MAX*_ was considered to correspond to appropriate balloon filling, with volumes below V_*MIN*_ denoting underfilling and volumes above V_*MAX*_ denoting overdistention. Within the appropriate filling range, we identified V_*BEST*_, i.e., the filling volume associated with the maximum difference between PesEI and PesEE. Pes: esophageal pressure. Data are shown as mean value ± standard deviation.

**FIGURE 2:**
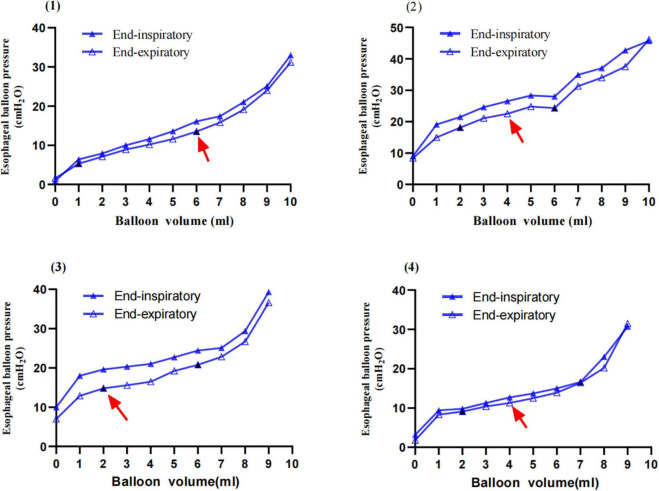
Examples of inspiratory and expiratory esophageal balloon pressure-volume curves. The solid blue triangle refers to end-inspiratory esophageal pressure (PesEI); the hollow blue triangle refers to end-expiratory esophageal pressure (PesEE). The solid black triangle refers to V_*MIN*_ and V_*MAX*_, and the red arrow represents the V_*BEST*_. (1) A 42-years-old female patient, BMI 21.5 kg/m^2^, with severe pneumonia (methicillin-sensitive Staphylococcus aureus, MSSA), pulmonary ARDS with focal lesion, VV-ECMO. PC 8 cm H_2_O, PEEP 10 cm H_2_O, TV 200.9 ml, Pplat 18 cm H_2_O, esophageal elastance 1.59 cm H_2_O/ml, and Pew at V_*BEST*_ 7.97 cm H_2_O; V_*MIN*_ 1 ml; V_*MAX*_ 6 ml; and V_*BEST*_ 6 ml. (2) An 18-years-old female patient, BMI 23.4 kg/m^2^, with infective endocarditis (IE), severe pneumonia (methicillin-resistant staphylococcus aureus, MRSA) and post-operative mitral valve replacement and ARDS with Diffuse lesion; PC 13 cm H_2_O, PEEP 8 cm H_2_O, TV 216.5 ml, Pplat 26 cm H_2_O; V_*MIN*_ 2 ml, V_*MAX*_ 6 ml, and V_*BEST*_ 4 ml, esophageal elastance 1.61 cm H_2_O/ml, and Pew at V_*BEST*_ 3.22 cm H_2_O. (3) A 29-years-old male patient, BMI 38 kg/m^2^, with severe acute pancreatitis (SAP), abdominal compartment syndrome (ACS), extrapulmonary ARDS with diffuse lesion, VV-ECMO; PC 10 cm H_2_O, PEEP 15 cm H_2_O, TV 475 ml, Pplat 25 cm H_2_O; V_*MIN*_ 2 ml, V_*MAX*_ 6 ml, and V_*BEST*_ 2 ml, esophageal elastance 1.56 cm H_2_O/ml, and Pew at V_*BEST*_ 0 cm H_2_O. (4) A 74-years-old male patient in the prone position, BMI 24.8 kg/m^2^, with severe pneumonia, septic shock, ARF with diffuse lesion; PC 9 cm H_2_O, PEEP 9 cm H_2_O, TV 358 ml, Pplat 18 cm H_2_O; V_*MIN*_ 2 ml, V_*MAX*_ 7 ml, and V_*BEST*_ 4 ml, esophageal elastance 1.41 cm H_2_O/ml, and Pew at V_*BEST*_ 2.81 cm H_2_O.

End-expiratory and end-inspiratory raw esophageal balloon P-V curves were obtained in all patients (Figure 1). An intermediate linear section was identified on the end-expiratory esophageal balloon P-V curve in each of the clinical measurements. The mean V_*MIN*_ and V_*MAX*_ were 2.17 ± 0.49 (range, 1.0–3.0 ml) and 6.79 ± 0.83 ml (range, 5.0–9.0 ml), respectively, while the best filling volume was 4.69 ± 0.16 ml (range, 2.0–8.0 ml). The slope of the linear section of the curve, i.e., Ees, was 1.35 ± 0.51 cm H_2_O/ml (range 0.26–2.38 cm H_2_O/ml). The estimated Pew at V_*BEST*_ was 3.16 ± 2.19 cm H_2_O (range, 0–7.97 cm H_2_O). Four different patients’ end-inspiratory and end-expiratory P-V curves are shown in Figure 2.

### Calibration procedure

**FIGURE 3:**
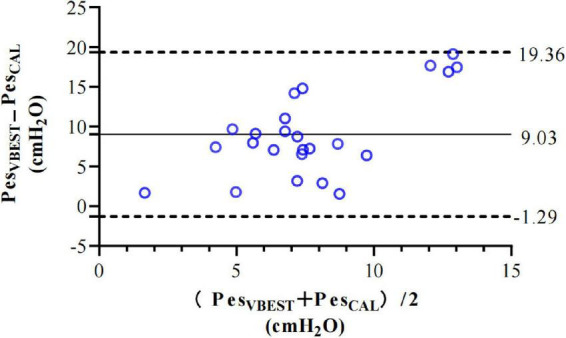
Bland-Altman limits of agreements analysis for Pes_*VBEST*_ and Pes_*CAL*_ at V_*BEST*_. Compared to Pes_*VBEST*_, bias (mean difference, continuous line) and precision (± 1.96 SD of the difference, dotted lines) of Pes_*VCAL*_ were 9.03 ± 10.33 cm H_2_O.

The calibration procedure is shown in Figure 3.

### Influence factors of the best esophageal balloon filling volume

**FIGURE 4:**
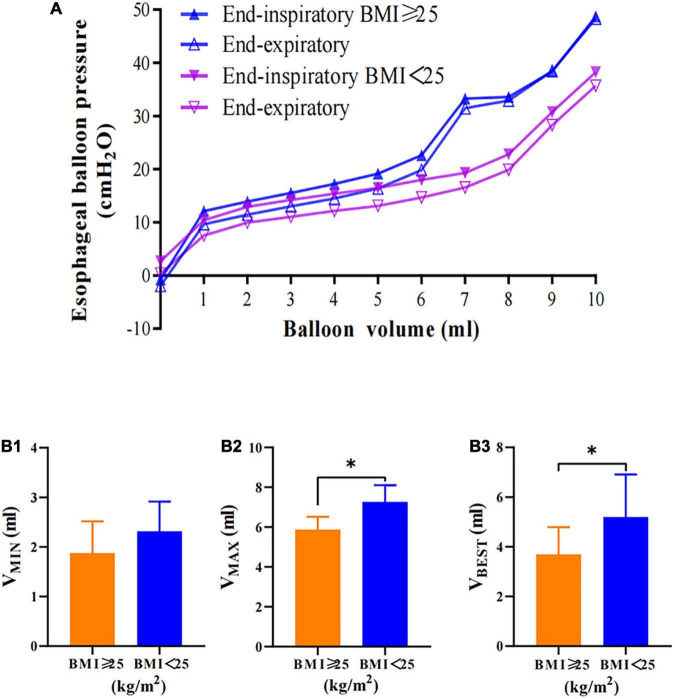
Esophageal balloon pressure-volume curves of different BMI groups. **(A)** Relationship between balloon filling volume and values of Pes in the BMI ≥ 25 kg/m^2^ (Pes_*EI*_, solid blue triangle; Pes_*EE*_, hollow blue triangle) and BMI < 25 kg/m^2^ (Pes_*EI*_, solid purple triangle; Pes_*EE*_, hollow purple triangle) groups. Patients in the BMI < 25 kg/m^2^ group had lower Pes than those in the BMI ≥ 25 kg/m^2^ group. **(B)** Patients in the BMI < 25 kg/m^2^ group had higher balloon volume than those in the BMI ≥ 25 kg/m^2^ group. *P < 0.05 compared with the BMI ≥ 25 kg/m^2^ group. Pes: esophageal pressure.

Patients with a BMI ≥25 kg/m^2^ had a lower V_*MIN*_ (1.88 [1.25–2.0] vs. 2.3 [2–3] ml, *p* = 0.11), a significantly lower V_*MAX*_ (5.88 [5.25–6] vs. 7.25 [7–8] ml, *p* = 0.006), and a significantly lower V_*BEST*_ (3.69 [2.5–4.38] vs. 5.19 [4–6] ml, *p* = 0.036) than patients with BMI < 25 kg/m^2^ (Figure 4).

**FIGURE 5:**
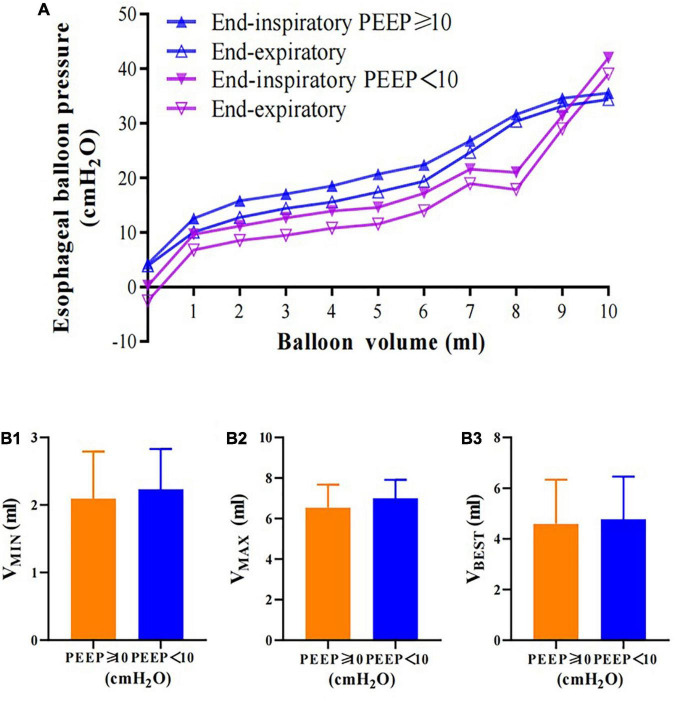
Esophageal balloon pressure-volume curves of different PEEP groups. **(A)** Relationship between balloon filling volume and values of Pes in the PEEP ≥ 10 cm H_2_O (Pes_*EI*_, solid blue triangle; Pes_*EE*_, hollow blue triangle) and PEEP < 10 cm H_2_O (Pes_*EI*_, solid purple triangle; Pes_*EE*_, hollow purple triangle). Patients in the PEEP < 10 cm H_2_O group had lower Pes than those the PEEP ≥ 10 cm H_2_O group. **(B)** Patients in the PEEP < 10 cm H_2_O group had higher V_*MIN*_, V_*MAX*_, and V_*BEST*_ than those in the PEEP ≥ 10 cm H_2_O group. Pes: esophageal pressure. No significant difference was observed.

Patients with PEEP ≥ 10 cm H_2_O had a lower V_*MIN*_ (2.09 [2.0–3.0] vs. 2.23 [2.0–3.0] ml, *p* = 0.6), V_*MAX*_ (6.55 [6.0–7.0] vs. 7 [6.5–8] ml, *p* = 0.29), and V_*BEST*_ (4.59 [4–6] vs. 4.77 [3.5–6] ml, *p* = 0.8) than patients with PEEP < 10 cm H_2_O (Figure 5).

**FIGURE 6:**
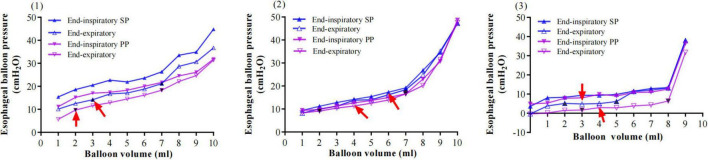
Examples of inspiratory and expiratory esophageal balloon pressure-volume curves in different positions. Three patients’ relationships between balloon filling volume and values of Pes in the supine position (Pes_*EI*_, solid blue triangle; Pes_*EE*_, hollow blue triangle) and the prone position (Pes_*EI*_, solid purple triangle; Pes_*EE*_, hollow purple triangle). The red arrow represents the V_*BEST*_, while the solid black triangle represents V_*MIN*_ and V_*MAX*_. Patients in the prone position had lower Pes than those in the supine position. (1) An 18-years-old female patient, BMI 23.4 kg/m^2^, with infective endocarditis (IE), severe pneumonia (methicillin-resistant staphylococcus aureus, MRSA), post-operative mitral valve replacement, and pulmonary ARDS with diffuse lesion. PC 13 cm H_2_O, PEEP 8 cm H_2_O; V_*MIN*_ 3 vs. 2 ml, V_*MAX*_ 6 vs. 6 ml and V_*BEST*_ 3 vs. 2 ml. (2) A 74-years-old male patient, BMI 24.8 kg/m^2^, with severe pneumonia, septic shock and pulmonary ARDS with diffuse lesions. PC 9 cm H_2_O, PEEP 9 cm H_2_O; V_*MIN*_ 2 vs. 2 ml, V_*MAX*_ 7 vs. 7 ml and V_*BEST*_ 6 vs. 4 ml. (3) A 74-years-old male patient, BMI 22.9 kg/m^2^, with severe pneumonia, and pulmonary ARDS with diffuse lesions. PC 12 cm H_2_O, PEEP 6 cm H_2_O; V_*MIN*_ 2 vs. 3 ml, V_*MAX*_ 8 vs. 8 ml and V_*BEST*_ 3 vs. 4 ml.

Patients in the supine position had a higher esophageal pressure than those in the prone position with the same balloon filling volume according to three patients’ data (Figure 6).

**FIGURE 7:**
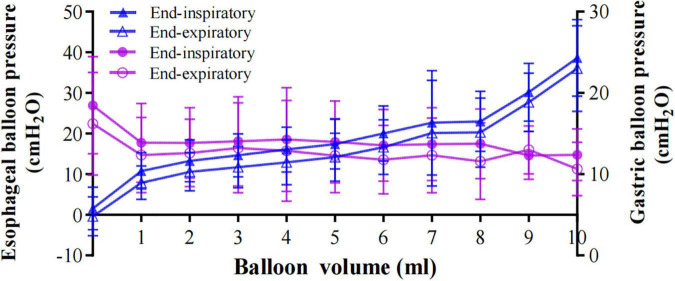
The relationship between gastric pressure and esophageal pressure. Solid blue triangles represent Pes_*EI*_ and hollow blue triangles represent *PesEE*. Gastric balloon pressure of end-inspiration (Pga_*EI*_) and end-expiration (Pga_*EE*_) are represented by solid purple circles and hollow purple circles, respectively. As the esophageal balloon volume increased, the gastric pressure remained relatively stable. Data are shown as the mean value ± standard deviation.

Moreover, the gastric pressure was relatively stable when the esophageal balloon filling volume increased (Figure 7).

## Discussion

We performed esophageal pressure monitoring in patients with ARF under invasive passive mechanical ventilation. We had three main findings: (1) The best filling volume (V_*BEST*_) can be quickly confirmed by the maximum esophageal pressure swing (ΔPes) of the intermediate linear section on the balloon P-V curve; (2) V_*BEST*_ varies widely in different patients or in different PEEPs, BMIs, and positions; and (3) Mindray catheters are suitable for esophageal manometry in critically ill patients. The gastric pressure was stable when the esophageal balloon filling volume increased.

There have been few clinical studies on esophageal balloon inflation pressure. Mojoli et al. monitored 36 patients under controlled ventilation with 50 series of esophageal pressure measurements. The esophageal pressure data were recorded from 0 to the maximum inflation volume recommended. The study showed that calibrated values of Pes are different from the absolute value of the esophageal pressure and the esophageal wall pressure value. The research steps are cumbersome, but it is of great significance for clinicians to accurately understand the esophageal pressure (9). Their research suggested that the intermediate linear section on the end-expiratory P-V curve was closely parallel to each other (*in vitro* and *in vivo*), when pressure generated by the esophageal wall was subtracted from Pes. Although the *in vitro* study showed that Pes is stable within the V_*WORK*_ on the P-V curve, the Pes *in vivo* linearly increased because of esophageal elastance (Ees) (9, 10, 12). Sun et al enrolled 40 patients under passive ventilation and verified the reliability of the method on a Cooper balloon catheter (geometric volume of 2.8 ml) (11). The method of calibrating Pes values was verified again during pressure support ventilation (15). Compared with the *in vitro* study, V_*BEST*_ was significantly increased in the *in vivo* test, suggesting that the pressure of the esophageal wall may have an effect on it. Mojoli et al. (9) showed that V_*MIN*_ is positively related to the surrounding pressure; that is, the greater the inflation volume is, the higher the esophageal pressure. Their research showed that the V_*BEST*_ was 3.5 ± 1.9 ml (range, 0.5–6.0 ml), which was larger than the traditional recommended small inflation volume. The study also found that the inflation volume that can pass the validation occlusion test is greater than 0.5 ml, so 0.5 ml may not be able to accurately assess the accurate value of the patient’s esophageal pressure. Sun et al showed that the V_*BEST*_ for smaller balloons (geometric volume of 2.8 ml) is 1.0 ml (range, 0.6–1.4 ml) and was highly variable among different patients and conditions (11). In this study, an esophageal pressure balloon with an inflation volume of 10 ml was used. V_*BEST*_ was 4.69 ± 1.36 ml (range, 2.0–8.0 ml), which was larger than that reported by Mojoli et al’s. Our study enrolled patients with lower PEEP and lower BMI compared with Mojoli’s research (9). Our study shows that this method should be used with different balloon catheters to determine the inflation volume for more accurate esophageal pressure measurement (Table 2).

**TABLE 2:** Balloon filling volumes in different esophageal catheters.

	Balloon volume (ml)	Vrec (ml)	V_*BEST*_ (ml)
NutriVent, Sidam, Mirandola, Italy	10	4.0	3.5 ± 1.9 (range, 0.5–6.0)
Cooper, LOT 177405, cooper surgical, United States	2.8	1.0	1.0 (range, 0.6–1.4)
Mindray, SDY-2, AMK Medical, Guangzhou, China	10	5.0	4.69 ± 1.36 (range, 2.0–8.0)

Vrec: factory-recommended inflating volume; V*_BEST_*: balloon volume with the largest difference between end-expiratory and end-inspiratory esophageal balloon pressure.

The intrathoracic pressure in critically ill patients can be affected by many factors, such as the accurate measurement of esophageal pressure. Therefore, the following factors may cause different measurement results. The first is body position. Previous studies have suggested that esophageal pressure when patients are in the supine position is higher than that in other positions due to the influence of mediastinal organs and tissues (16, 17). Washko et al. (18) showed that higher Pes in the supine position can be affected by a direct compression artifact and the change in lung relaxation volume in different positions. Yoshida’s study in an animal model and human cadavers showed that esophageal pressure measurement in the supine position as a substitute for Ppl at the mid-chest is suitable (19). Previous research was performed with patients in different body positions (supine with the bed at 20–30 degrees head up, lateral and prone at 45 degrees), while our study was performed with patients in the supine position or prone position without elevating the head of the bed. Our research showed the same results as other studies that esophageal pressure was higher in the supine position than in the prone position (Figure 6).

Our findings also showed that the higher the BMI and PEEP value were, the higher the esophageal pressure was (Figures 4, 5). Although previous studies (9) have suggested that, at increasing Pes, filling volumes should increase, our results on patients with higher PEEP and higher BMI (both having higher Pes) do not confirm such a finding; indeed, filling volumes were, overall, not significantly different from patients with lower PEEP and lower BMI, respectively. This suggests that more work is required in this regard. Next, patients in this study had more severe conditions with higher APACHE II scores and SOFA scores. The same patients at different disease stages had different V_*BEST*_ values ranging from 3 to 6 ml. Finally, the V_*BEST*_ was catheter-specific, such as in NutriVent and Marquat catheters, even at the same catheter volume, in an *in vitro* study (12). Similar conclusions may be drawn in *in vivo* tests. The results confirmed the accuracy of the current method used to determine V_*BEST*_. Therefore, the filling volume of the esophageal balloon catheter should be rechecked for more accurate results when the factors that may affect esophageal pressure change.

Hence, from the perspective of overall evaluation, the vital contribution to this study was that Mindray’s esophageal pressure catheters can be used to guide personalized lung protection strategies. The esophageal pressure will increase as the esophageal balloon volume increases *in vivo* and we should calibrate the esophageal pressure accordingly. In addition, we also found that different positions and different BMI and PEEP values affected the optimal inflation value. We recommend that when we perform esophageal pressure monitoring, the optimal filling volume of Pes should be routinely reselected to clarify the exact esophageal pressure, and calibration of esophageal pressure is also needed.

It shows that there is an overall bias of 10 cm H_2_O between raw and calibrated Pes at V_*BEST*_, with large limits of agreement (Figure 3). This is higher than Mojoli and Sun’s study, showing that the esophageal wall pressure leads to an overestimation of Pes around 10.33 cm H_2_O (9, 11). The esophageal pressure catheter used in our study is different from other studies (7). Some of the included patients are either post-thoracotomy or have focal lung involvement disease. The effects of PEEP, BMI, and position on patients require further study. This may be the reason why our study is different from other studies.

To best of our knowledge, this is the first study to use esophageal and gastric balloon catheters to titrate the proper esophageal balloon filling volume and clarify the relationship between esophageal pressure and intragastric pressure. Diaphragmatic pressure (Pdi) can be used to assess breathing, respiratory muscle function, and the presence of diaphragm paralysis (20). It is well known that Pdi can be calculated by Pga minus Pes. Our research showed that the gastric pressure (Pga) is relatively stable, while Pes increased with increased balloon filling volume. So, the value of Pes can have an effect on Pdi.

## Limitations of the study

In this study, the supine and prone positions were used to measure esophageal pressure, and the effect of different body positions on esophageal pressure is still inconclusive. This study, based on the method of Mojoli et al. (9), used the calibrated values of Pes to minimize possible factors affecting the accuracy of esophageal pressure and requires further evaluation. The research was performed with only one Mindray esophageal balloon catheter. Although our research and previous studies have confirmed the reliability of the method (9, 11, 15), whether the results can be directly generalized to other esophageal catheters and other patient populations is still in question. The sample size of this study was small, and one patient underwent up to five tests, which may be a factor that led to the overall higher esophageal pressure values in this study than in other studies (9, 11). The esophageal pressure mainly indicates the intrathoracic pressure in the middle of the lung, and whether it can be used to assess post-thoracotomy or focal lung disease needs more research to be confirmed.

## Conclusion

In summary, the method to identify the best filling volume of esophageal balloon catheters is feasible. This approach is well validated in Mindary’s intercropping. It might aid in developing personalized mechanical ventilation at bedside, such as different body positions and different intervention methods. Further study is required to validate the clinical applicability of this method.

## Data availability statement

The datasets used or analyzed in this study are available from the corresponding authors on reasonable request.

## Ethics statement

The studies involving human participants were reviewed and approved by the Institutional Research and Ethics Committee of the Peking Union Medical College Hospital (NO. ZS-2458). The patients/participants provided their written informed consent to participate in this study.

## Author contributions

YL and HH took responsibility for the integrity of the study as a whole. JJ was responsible for the collection of data, data management, statistical analysis, and drafted the manuscript. LS was responsible for the study design, conception, data management, and statistical analysis. WC and CW participated in the collection of data and data management. XR, BT, and HZ took responsibility for the study design and conception. All authors revised the manuscript for content and contributed to the article and approved the submitted version.
